# Common somatic symptoms, causal attributions of somatic symptoms and psychiatric morbidity in a cross-sectional community study in Santiago, Chile

**DOI:** 10.1186/1756-0500-4-155

**Published:** 2011-05-26

**Authors:** Petros Skapinakis, Ricardo Araya

**Affiliations:** 1 Department of Psychiatry, University of Ioannina School of Medicine, 451 10 Ioannina, Greece; 2 Department of Psychiatry, University of Bristol, Oakfield House, Oakfield Grove, Bristol BS8 2BN, UK

## Abstract

**Background:**

It has been suggested that individuals from non-western countries tend to deny or mask psychological symptoms of common mental disorders and to present with somatic symptoms. The aim of the present paper was to investigate the association between common mental disorders and somatic symptoms in a representative sample of the general population of Santiago, Chile

**Findings:**

This was a cross-sectional study of a stratified random sample of 3807 subjects living in private households in Santiago, Chile. Psychiatric disorders were assessed with the revised Clinical Interview Schedule. We found a strong association between the presence of somatic symptoms and psychiatric disorders (odds ratio 3.20, [95% confidence interval 2.52 - 4.05]). In addition, subjects who attributed their somatic symptoms to psychological or mixed psychological/physical causes were more likely to be cases compared to subjects who made physical attributions only (odds ratios 7.10 [95% CI 4.49-11.25] and 9.27 [6.00-14.34] respectively.

**Conclusions:**

The study confirms previous observations from more selected samples that subjects of Hispanic origin are generally aware of the link between somatic symptoms and psychological ill-health and do not hide or "mask" their psychological symptoms.

## Background

The experience of somatic symptoms is very common in the general population [1]. Several studies, both from the developed [2-5] and developing world [6-8] have shown that musculoskeletal pain, back pain, headache, respiratory symptoms, fatigue and dizziness are the symptoms most often reported by unselected samples of the general population or primary care attenders. These symptoms are usually benign and self-limited and only rarely do they lead to medical consultation [2].

The strong association between somatic symptoms and the common mental disorders of depression or anxiety has been reported by many studies in the past [9-12]. It has been argued that somatic and psychiatric comorbidity is a key factor in one's decision to consult a general practitioner for a health problem [13,14]. In addition somatic symptoms, especially of pain or fatigue, are common manifestations of depression or anxiety disorders. In primary care, patients with psychiatric disorders often present with somatic rather than psychological symptoms [14], and this may reduce the general practitioners' ability to recognize and treat depression or anxiety disorders [15,16]. The way individuals interpret their somatic symptoms, the so called causal attributions of somatic symptoms [17], influence the decision to consult and the ability of the doctor to recognize any psychiatric condition [18]. Individuals who interpret their symptoms as being due to a physical problem are more likely to consult a doctor and less likely to uncover psychological symptoms, making recognition of depression or anxiety more difficult [19].

Although the association between somatic symptoms and psychiatric disorders has been reported in all cultures [6], there has been a long-standing assumption that it is stronger among individuals from the developing world, or in specific cultures especially of Hispanic origin [5]. This association has also been reported in primary or tertiary care centres [7,14,20]. This finding has often been interpreted as evidence that non-western patients are more likely to "mask"or "deny" psychological symptoms of depression or anxiety disorders and tend to focus on the somatic aspects of these illnesses [21,22]. An international study in primary care that investigated this issue could not find evidence of denial, but did report a tendency for over-reporting of somatic symptoms in general practitioners, mainly influenced by the patients'expectations of the doctor-patient relationship in various settings across the world [14]. Studies of unselected samples of the population have mainly investigated whether there is an association between somatic symptoms and psych disorders and did not attempt to interpret this association in terms of the causal attributions of the somatic symptoms [5,9]. Moreover, studies using representative samples of the general population from non-western countries are scarce [6]. In this paper we report data from the Santiago mental health survey [23]. Our aim was to investigate the association between somatic symptoms and common mental disorders in a representative sample of the general population of Santiago. We also examined causal attributions of somatic symptoms and we hypothesized that subjects making psychological attributions would be more likely to be cases of common mental disorders, i.e. that subjects would be aware of the connection between somatic symptoms and psychological distress.

## Methods

This paper describes data from the Santiago Mental Disorders Survey undertaken between 1996 and 1998 in Santiago, Chile [23]. The sampling involved a three stage clustered design. Households within 200 sectors from all the 35 boroughs of Santiago, capital of Chile, were randomly chosen with a probability proportional to the population size. A larger sampling fraction was needed in the most affluent boroughs to permit testing for socioeconomic differences between groups. One person per household was chosen at random using the Kish method. The sampling framework was the total adult population living in private households of Santiago, representing 3 217 177 people at the time of the field work. Interviewers were instructed to make at least four visits before declaring an address as lost. Household size was defined as the number of people aged 16-64 years residing in that property who were eligible for interview. The selected households were not visited before initiation of the fieldwork, so it was not possible to ascertain a priori if any of the residents did not meet the criteria for inclusion in the study.

The sample framework comprised 4693 addresses, 393 of which were declared unusable because they were nonresidential or contained only residents over 65. So effectively 4300 private households were approached. Altogether 3870 subjects were interviewed, a response rate of 90%. More details on the methodology are given elsewhere [23].

### Measurement of somatic symptoms and their causal attributions

All participants were asked the screening question "Did you experience any of the following symptoms during the last 7 days?". A card with the following somatic symptoms was shown and read out aloud: Headache, backache, tiredness or fatigue, stomach pain or discomfort, chest pain or discomfort. If symptoms were present, individuals were asked to identify the two most important ones and to estimate their intensity (little, moderate, severe) and frequency (once, two to three times, everyday, permanently).

Subjects who had experienced any somatic symptom of at least moderate intensity for two or more days during the last seven days were considered as having "somatic symptoms". It should be noted that this definition did not take into account whether the symptom was medically "unexplained" or not.

Subjects who had experienced somatic symptoms were also asked what might have caused their symptoms. The four alternatives were: a) a physical illness, b) a mental health problem, c) both, or d) other causes (e.g. working conditions, religious issues and so on)

We combined answers to these two questions on somatic symptoms and their causal attributions in order to further classify the participants into five categories: no somatic symptoms (reference group), somatic symptoms with physical attributions, somatic symptoms with psychological attributions, somatic symptoms with mixed attributions (both physical and psychological), somatic symptoms with other attributions.

### Measurement of Psychiatric Morbidity

Psychiatric morbidity was assessed with the Revised Clinical Interview Schedule (CIS-R), a standardized interview administered by trained lay interviewers. The English and Spanish versions of the CIS-R have been used extensively in community and primary care studies [24]. A score on the CIS-R of 12 or more has been used in the past to define cases of common mental disorders. Using this threshold about 25% of the total sample would be classified as a case of common mental disorder. However, because fatigue and somatic symptoms such as pain contribute to the total CIS-R score, we excluded these two sections from the total scores. Thus we defined as cases of common mental disorders all those scoring above the 75^th ^percentile of the total corrected score in order to obtain a comparable prevalence. The case threshold obtained this way had a value of 10 or more points.

### Other Variables

We collected information on the following sociodemographic variables: Sex, age, educational status and marital status.

### Statistical Analysis

Statistical analysis was carried out with the survey commands of the program STATA that take into account the complex sampling design and weights applied to the original data [25]. The association between psychiatric morbidity (used as the dependent variable) and somatic symptoms (and causal attributions of somatic symptoms) was examined in a weighted logistic regression model. We report odds ratios with 95% confidence intervals, adjusted for sociodemographic variables.

## Results

The mean age of the sample was 35.6 (standard deviation 14.11), 53% were women, 55% were married and 54% had completed secondary education.

There were 846 cases of psychiatric morbidity (prevalence 24.4%, 95% CI 22.2 - 26.6). A total of 1370 subjects reported a somatic symptom that met our criteria of frequency and intensity (prevalence 37.2%, 95% CI 35.1 - 39.4). The most common symptom reported was headache (49% of subjects with somatic symptoms), followed by backache (23%) and fatigue (18%). Causal attributions of somatic symptoms were more often of a physical nature. Almost one third of the subjects with somatic symptoms (n = 1370) made psychological explanations to interpret their somatic symptoms while in the subgroup of subjects with both psychiatric morbidity and somatic symptoms (n = 497) this percentage rose approximately to one half (Table 1).

**Table 1:** Causal attributions of somatic symptoms in the general population of Santiago, Chile.

Causal attributions of somatic symptoms	**In subjects with somatic symptoms only (n = 1370**^**1**^**)**	In subjects with somatic symptoms who were also cases of psychiatric morbidity (n = 497)
Physical	58.2%^2 ^(54.3 - 62.0)	43.3% (37.2 - 49.6)
Psychological	10.8% (9.0 - 13.0)	16.6% (12.5 - 21.9)
Physical and Psychological	18.4% (15.6 - 21.6)	30.7% (24.8 - 37.5)
Other	12.6% (10.1 - 15.5)	9.3% (5.7 - 14.7)

1. From the total sample of 3870 participants, 1370 reported at least one somatic symptom (weighted prevalence 37%) and 2500 did not report any somatic symptom. Please see text (results section) for details.

2. Actual number of subjects. Percentages are weighted to reflect the stratified sampling procedure and non-response.

Subjects with somatic symptoms were almost three times more likely to be psychiatric cases (odds ratio: 3.20, 95% CI 2.52 - 4.05). Moreover, subjects with psychological or mixed attributions had the highest likelihood of being psychiatric cases compared to subjects without somatic symptoms (Table 2). Compared to subjects with somatic symptoms and physical attributions, subjects with psychological or mixed attributions were significantly more likely to be psychiatric cases (p < 0.001 for both comparisons)

**Table 2:** Association between causal attributions for somatic symptoms and psychiatric morbidity in the general population of Santiago, Chile.

Logistic Regression analysis (all subjects, N = 3870)	**Number (%)**^**1 **^**of subjects with psychiatric morbidity**^**2**^	**Odds Ratios**^**3 **^**(95% CI) of being a case of psychiatric morbidity**^**2**^
**No somatic symptoms**	349/2500 (15.6%)	1.00 (reference)
Somatic symptoms and physical attributions	221/740 (29.2%)	2.08 (1.57 - 2.75)
Somatic Symptoms and psychological attributions	87/170 (60.2%)	7.10 (4.49 - 11.25)
Somatic symptoms with mixed attributions	140/249 (65.4%)	9.27 (6.00 - 14.34)
Somatic symptoms with other attributions	49/211(29.0%)	1.98 (1.19 - 3.27)

**All somatic symptoms combined **(compared to no somatic symptoms)	497/1370 (39.2%)	3.20 (2.52 - 4.05)

1. Actual number of subjects. Percentages are weighted to reflect the stratified sampling procedure and non-response.

2. Psychiatric Morbidity assessed with the revised Clinical Interview Schedule (CIS-R).

3. Odds ratios adjusted for age, sex, educational status and marital status.

## Discussion

In this cross-sectional community study in Santiago, Chile we found a strong association between having common somatic symptoms and psychiatric morbidity as measured by a standardized psychiatric interview. This association was stronger in subjects making psychological or mixed (psychological and physical) attributions about their somatic symptoms compared to those making physical attributions only or those not having somatic symptoms at all.

Certain limitations should be considered before interpreting these results. First, we were unable to distinguish between medically explained or unexplained symptoms. Since past research has shown that the association of psychiatric morbidity with somatic symptoms is even stronger when medically unexplained symptoms are only considered [26], we think that this limitation does not threaten the validity of our results. Second, we measured attributions in a rather crude way by asking subjects to answer to a simple question on what caused their symptoms, something we had used successfully in our study in primary care [16]. Third, this was a secondary analysis of a data set that was not specifically designed to test the hypotheses of the study. In addition, the study was carried out between 1996-1998 and it is possible that political, economical and social changes in Chile might have an effect in the way people express their symptoms and ask for help. We would like to note however, that during the same period, Chile is considered a relatively stable middle economy in Latin America with steady growth and an established democratic regimen.

Psychological distress often presents with somatic symptoms. Previous research had argued that patients of Hispanic or Asian origin had a tendency to deny psychological attributions to somatic symptoms and/or express their psychological distress in somatic terms [11,21,22]. Psychiatrists have been advised to take these cultural factors into account when making the diagnosis of depression [27]. However, this and other studies show that this assumption is no longer tenable [14]. In our study, subjects did not show evidence of denial of their psychological distress and they were well aware that their psychological distress might have contributed to their somatic symptoms. Thus, we confirm in this representative and randomly selected community sample our previous finding in primary care that Chilean people are equally likely than individuals from the more developed world to appreciate the true nature of their health problems [16]. Selection bias may explain the previous reported findings that subjects from non-western samples may deny psychological symptoms and present with somatic symptoms. It is very likely that subjects with both somatic symptoms and psychiatric disorders are more likely to consult a general practitioner compared to subjects with psychological symptoms only, especially in less developed countries as the WHO study of mental illness in primary care has shown [14]. In any case, directly enquiring about the causal attributions of somatic symptoms may be a simple and useful way to identify and recognize the so-called hidden psychiatric morbidity in these settings.

## Competing interests

The authors declare that they have no competing interests.

## Authors' contributions

PS carried out the analysis and drafted the paper. RA was responsible for the conception of the study, designed and supervised the data collection of the original survey, helped in data analysis and interpretation of the results. Both authors read and approved the final manuscript.
